# Supplementary material for: Beverage Intake and Drinking Patterns—Clues to Support Older People Living in Long-Term Care to Drink Well: DRIE and FISE Studies
Source: Nutrients. 2019 Feb 21;11(2):447. doi: 10.3390/nu11020447 (PMC6412353; doi:10.3390/nu11020447)
Supplement: Supplementary file 1 [file nutrients-11-00447-s001.pdf]

# Supplementary materials for Jimoh et al, 'Beverage intake and drinking patterns – clues to support older people living in long-term care to drink well: DRIE and FISE studies'

## Results section in greater detail

*3.2 Quantity of fluid consumed*

DRIE did not quantify fluid intakes, though it assessed hydration status through directly measured serum osmolality, while FISE did not re-measure serum osmolality but comprehensively assessed fluid intake. As previously reported, 20% of the 188 DRIE participants were dehydrated (serum osmolality >300mOsm/kg), 28% had impending dehydration (295 to 300mOsm/kg), while 52% were well hydrated (serum osmolality 275 to <295mOsm/kg ) [3]. The 22 FISE participants were similar (18% dehydrated, 59% well hydrated) during their DRIE interviews, but hydration status was not reassessed at FISE interview, usually several months later. FISE participants' mean total drinks intake was 1787ml/day (SD693, range 810-3403), and did not differ significantly by sex (2034ml/day SD843 for men, 1748ml/day SD684 for women). Twelve of 22 (55%) FISE participants achieved EFSA drinks goals (3/6 of men drank ≥2.0L/d and 9/16 of women drank ≥1.6L/d).

*3.3 Types of drinks enjoyed by residents and provided by long-term care facilities*

DRIE participants were asked "What are your favorite drinks?" and all replies collated, with participants prompted for non-alcoholic beverages where only alcoholic drinks were mentioned. Drinks preferences were expressed by 174 residents (of whom 11 stated "no preference", 14 did not reply, and 163 expressed one or more preferences). Tea and coffee were most popular (named by 44% and 21%, respectively), while fruit juice, water, squash or an alcoholic drink were each expressed as favorite drinks by >10% participants (Figure 2). These preferences were reflected in drinks provided by care homes, with ~90% of residents being provided with a cup of tea at some point during each day according to both DRIE (resident-reported, 90%) and FISE (directly observed, 91%) data. Mean FISE intake was 690ml of tea/participant/day. Coffee was provided daily to 51% of residents. Water, squash, fruit juice, and hot milky drinks were offered to over a third of residents on most days. Milk, carbonated drinks, Bovril and Oxo, alcoholic drinks and other drinks were regularly provided to fewer residents (Figure 2). FISE direct observation showed that tea, water and coffee together contributed 80% (38%, 27% and 15% respectively) of total beverage intake. Fruit juice, hot milky drinks, milk, alcohol and supplements each accounted for ≤6% intake.

FISE participants who met EFSA minimum intakes drank significantly more water (p=0.002) than those who did not, other drink types were not significantly different. Total drinks intake was moderately but significantly correlated with water (r=0.5, p=0.011) and milk (r=0.6, p=0.006) but not tea (r=0.4, p=0.07), coffee (r=0.4, p=0.08) or squash (r=0.05, p=0.84). Water is the drink which residents were most able to access independently.

103 DRIE participants replied to "What drinks do you like that you don't get here, at this care home?" and 75 (73%) reported they did not miss any drinks (4 added they had personal drinks supplies, sometimes brought by relatives). Twenty eight (27%) did report missing some drinks, usually stating a specific drink or drinks. Alcoholic drinks (10 participants), different or better-quality tea or coffee (8 participants, "a good cup" or "real"), and a variety of non-alcoholic drinks (14, including 2 "own" soup, 3 fruit juice, 1 hot milk, 2 milkshakes, 1 Oxo, 1 soda water, 1 lemonade, 1 cod liver oil and honey and 2 "good" or "iced" water) were missed.

*3.4 Finishing drinks*

DRIE researchers observed a drink provided to residents during, before or after the DRIE interview or during a meal. For 101 participants we were able to observe for ≥30 minutes. 73% were completely consumed within 30 minutes (18% within 5 minutes), 22% were not finished, but more than half was drunk by 30 minutes, and 5% were not drunk at all, or less than half was consumed. This was often in the presence of a researcher, who may have had a drink at the same time - residents may drink more when in a more social situation [5] or conversely drink more when not being distracted by an interview.

FISE Drinks Diaries (self-reported) recorded which and what proportion of drinks were consumed. Drinks Diary data showed that 86% of cups of tea and 85% of coffee served were drunk completely (finished), while 42% of glasses of water and 14% of squash were finished. All fruit juice (18/18 drinks), hot milky drinks (9/9) and alcoholic drinks (3/3) provided were completely consumed.

*3.5 Numbers and timing of drinks - routines*

DRIE participants were asked about their normal drinks pattern, 179 replied. The mean number of self-reported drinks per day was 8.0 (range 1-16). However, direct observation of FISE participants, likely to be more accurate, suggested a mean of 11.4 (SD2.9) drinks/day (range 6-16). The number of drinks/day of those who met the EFSA standard (12.8 SD2.4 drinks/day, range 9-16) was significantly higher ($p=0.008$) than those who did not (9.5 SD2.5, range 6-15). Number of drinks/day correlated significantly and positively with total drinks intake ($r=0.642$, $p=0.001$).

Care home staff were asked about number and timing of drink offers to DRIE residents (Figure 3). Numbers of drinks offered peaked at mealtimes, and were lowest at night, overall 8.8 drinks/day offered. As not all drinks offered were consumed, and drinking 8.8 drinks/day was likely to be insufficient (if provided in 150ml cups total intake would be 1.3L/d) residents often had to help themselves to additional drinks to drink enough.

Drinks observed during DRIE were largely presented in cups with saucers (45%, volume ~150ml), with 18% in mugs (~225ml), 22% in glasses (volumes vary), 10% in specialist drinks containers (such as spouted mugs), and 5% in a teapot. Drinks directly observed during FISE were often presented in cups with saucers (32%, ~150ml), 3% in small mugs (~180 ml), 17% big mugs (~250ml), 20% small glasses (~140ml), 15% bigger glasses (~260ml), 4% a teapot or cafetiere, 4% jugs (usually water or squash) and 5% in other vessels including drinking aids, plastic cups, cans and wine glasses. Where drinks were provided in small cups or glasses (volume ~150ml) then 11 drinks would be needed to provide 1.6L/d required by women, and 14 drinks by men (assuming all drinks were completely consumed and cups well-filled).

Whilst formal drink offers tended to be at mealtimes, as reflected by residents self-reported drinking times (Figure 4), FISE found that beverage intakes were greater at non-meal times. Direct observation showed that 59% of drinks volume was taken between meals (mean 1047ml/d, SD433) with significantly less, 41% (mean 740ml/day, SD298, $p=0.009$) taken at meal times. This pattern was consistent for those who met the EFSA drinks intake standards and those who did not, irrespective of gender.

Drinks taken with medication (part of non-meal drinks intake) accounted for 10% of total drinks intake; ranging from 7 to 676ml/24-hours (Figure 5). Over 10% of drinks volume was consumed at each meal, and mid-morning, mid-afternoon and evening drinks rounds. Intake over-night (4%) and between waking and breakfast (5%, when residents tell us they are most keen to drink) were minimal (Figure 5).

FISE participants' drinks intake over 24-hours of observation is presented in Figure 6, by whether participants met EFSA drinks intake recommendations or not. Drinks intake peaked at meals and drinks trolley rounds, suggesting that residents were largely dependent on care staff for drinks. FISE participants who met EFSA drinks intake standards had more to drink on average (although not

statistically significantly more) at almost every drinking occasion through the day, especially overnight p=0.14), before breakfast (p=0.06) and with medications (p=0.05, Figure 6). To obtain this additional fluid they are likely to have had to help themselves to some drinks.

We asked DRIE participants "Do you always have a drink when you wake up?" (recorded as "yes always", "usually" or "no never"), followed by "Do you always have a drink at breakfast?" and so on through the day. Over 80% replied "yes always" for drinks at breakfast, lunch and the evening meal, closely followed by drinks in mid-morning and mid-afternoon. Less than 60% of participants stated they always had a drink on waking or during the evening, and most stated they never drank through the night. Residents who reported always or usually drinking on a particular occasion generally had a single drink, though having more than one drink was most common at breakfast, when the mean number of drinks was almost 1.5. Residents who answered "usually" to taking drinks on a specific occasion sometimes noted drinks rounds were occasionally missed by staff, or the resident would be omitted from the round (perhaps because they were not in a social space within the home, or went out of the home during the drinks period). Drinks provision between waking and breakfast (provided when staff support residents to get up, wash and dress, several hours long for those who woke early) was often described as depending on which staff were working that morning.

*3.6 Variety of drinks*

Number of types of drinks/day is a measure of variety. A person provided with two cups of tea, two cups of coffee, an Ovaltine and an orange juice was counted as having six drinks, but four types of drink (tea, coffee, Ovaltine, orange juice). Mean intake was 3.5 different drink types/day (SD1.3, range 1-7) by self-report in DRIE, while close observation in FISE suggested 4.0 drink types/day (SD1.3, range 2-7). Variety did not differ significantly between those who met EFSA guidance (mean 4.0 drink types/day, SD1.2) and those who did not (mean 3.8, SD1.3) in FISE (r=0.4, p=0.10).

*3.7 Thirst and knowledge of hydration status*

DRIE participants were asked whether they thought they drank enough to keep healthy. Responses were recorded for only 52 residents, of whom 54% replied "yes", 33% "no" and 14% were unsure. Belief in drinking enough, or not, was not correlated to hydration status. Just before their blood test they were asked "are you currently feeling thirsty?". While 50 (27%) answered "yes" (the remaining 138 answered "no") there was no relationship between thirst and hydration status as assessed by serum osmolality (p = 0.998) [3]. Thirst is not a good guide to the need to drink in older adults.

Care home staff were asked whether DRIE participants were likely to be at risk of dehydration and whether they required help with drinking. Staff reported that 3% (n=6) of residents required help with drinking, but they felt a quarter of DRIE participants (n=46) were at risk of dehydration. Dehydration risk factors they highlighted were largely medical limitations or the need for prompting residents to drink. When DRIE participants were asked "Do you have any problems swallowing?" 17% (n=34) reported "yes", while 5% (8 of 165) were prescribed thickened drinks. This may suggest swallowing problems are underdiagnosed/under-treated but further investigation is needed. There was no relationship between staff reported risk of dehydration or needing help with drinking and actual dehydration [3]. DRIE data suggested reduced cognition was significantly correlated with greater serum osmolality and higher dehydration risk in univariate and multivariate regression [3]. This was echoed in FISE, where cognitive status (by MMSE score) was significantly correlated with drinks intake (r=0.511, p=0.015 by Pearson correlation).

*3.8 Obtaining drinks outside routine provision*

Three quarters of DRIE care homes reported that residents could help themselves to drinks if wanted, one quarter did not, though all homes stated residents could ask staff for drinks when wanted. When DRIE residents were asked what they would do if they felt thirsty 54% of the 171 who replied said they would help themselves to a drink. Sometimes it was indicated that this would come from a

jug of water or squash in their bedroom, a private supply of bottled or canned drinks, or from the bathroom tap. 15% reported they would ask a member of staff for a drink, 15% said they would either get their own drink or ask, 8% reported they would wait until the next drinks round or meal, 6% gave non-specific answers such as “drink”, while 2% said that they didn’t get thirsty or worry about drinking. Sometimes it was stated that asking staff would depend on which staff were on duty. FISE observations suggested that residents rarely asked for, or helped themselves to, drinks.

*3.9 Reasons for cutting down on drinks*

A third (32%, n=64) of DRIE participants responded to the question “Do you always drink as much as you would like to?” with “no”. Some residents gave specific reasons for this including: concerns around getting to the toilet (n=14); lack of available drinks (n=6); not being interested in drinking (n=5); not liking drinks provided (n=4); functional drinking problems (n=3); forgetting (n=3); and being reluctant to ask staff for drinks (n=1). When DRIE participants were (later) asked “Do you ever drink less so you won’t need to get up for the toilet in the night?” 17% (n=34) reported they sometimes drank less for this reason. When asked “Do you ever worry that you won’t be able to get to the toilet to pass urine in time?” a quarter of DRIE participants (n=51) reported they did worry, and 8% (n=15) reported drinking less because of this. These data confirm that worries about incontinence and toilet-related incidents can lead to reduced drinking in older adults [17].
